# After the Hype, HOPE (and HPS): some lessons from the Women's Health Initiative Trial, the Heart Outcomes Prevention Evaluation Study and the Heart Prevention Study

**DOI:** 10.1590/S1516-31802003000100001

**Published:** 2003-01-02

The importance of the results from the "Women's Health Initiative" (WHI) has perhaps not yet been fully comprehended by doctors and public health professionals. There has been a lot of criticism about the hormone formulations, entry age for the study, lower absolute risk of coronary disease and cancer, biological plausibility, benefits in relation to osteoporosis, and potential bias due to subclinical atherosclerosis.

Unfortunately, we doctors are more and more disposed to disseminate information through the press in an imprecise and sometimes vulgar manner. We just have to recall how many colleagues have been appearing in all the media advising all menopausal women to seek out a gynecologist in order to have hormone replacement. This behavior continues to prevail in other specialties too, and it has serious ethical implications. We have a duty to no longer accept such abusive personalistic appearances in the media. Such attitudes transform the noble art of our "confessional consultation office" into a media show that resembles those of the "electronic ministers of religion".

The second point extrapolated from discussion of the WHI is in relation to primary prevention, whether this relates to cancer, coronary disease or dementia. The doctor's responsibility when proposing an untested intervention for healthy individuals is much greater than in the prescription of medications or other types of therapeutic intervention that we seek out to mitigate pain and suffering. I will cite the example of beta-carotene administration for the prevention of lung cancer. Smokers, who ought to be the ones getting greatest benefit from this, have presented a paradoxical increase in the incidence of and mortality from lung cancer.^1^ Because of this, it is not admissible to utilize the argument that is spread around in favor of hormone replacement therapy (HRT), in which the absolute risk of myocardial infarct and breast cancer is small. In primary prevention, there is no such thing as a "small" risk, or a large one! Any risk represents a contraindication for adoption, as can be seen in the precautions and the time required for vaccines to be approved for use by the general public.

The third point is in relation to biological plausibility, such as suggested in the Bradford-Hill rules of causation.^2^ This is the great drama of the present moment. There is no point in having medications at our disposal that alter or modify biological variables if they are inefficacious in reducing clinical events. The case of hormone replacement is not the first disaster described. This editorialist, in the initial phase of his professorial career, gave several seminars and classes about myocardial infarct treatment, in which there was a discussion of an article published in the *New England Journal of Medicine*^3^ with an editorial by Bernard Lown^4^, and also a case-control study by Alvan Feinstein^5^ (ironically, one of the creators of clinical epidemiology, the precursor of evidence-based medicine). In these, it was shown that lidocaine was a medication for immediate use that was fundamental in attending to patients with myocardial infarct, because of its action in suppressing ventricular arrhythmia, which is the main cause of early death in myocardial infarct. Much later, it was surprisingly shown than lidocaine was, despite reducing ventricular arrhythmia, increasing the mortality rate!^4-8^ How many deaths did we induce? I think that clinicians learnt a lot from this error of serious proportions (there has been an anecdotal calculation showing that there were more deaths through the use of lidocaine than of American soldiers in the Vietnam war!).

Moreover, regarding biological plausibility, despite the effects of HRT in reducing the high-density lipoprotein (HDL) fraction of cholesterol, among other effects, the information showing that the estrogen-progestin combination increases the levels of ultrasensitive C-reactive protein deserves to be highlighted, since this is a marker for the future development of coronary disease.^9^

The fourth point relates to the type of medication and its administration route. This has been exhaustively debated and may present conflicts of interest among the debaters. The use of captopril or enalapril for preventing cardiovascular events has been discussed as a substitute for ramipril, which was utilized in the Heart Outcomes Prevention Evaluation (HOPE) study.^11^ In the same way, there has been discussion of whether atorvastatin would present the same risks as cerivastatin for developing myopathy. The argument always favors the party that has an interest in the prescription. In the case of HRT, I would argue that the proposal for medication, dose and means of administration that were tested in the Women's Health Initiative was the same as was utilized in observational studies that have stimulated the use of HRT. Thus, there is a need for historical coherence in present-day criticism. The possibility exists that there may have been "enormous misfortune" in the choice of the "only" risk formulation. This has already taken place in other situations, and the solution will come over the long term. One example is the delay in the use of proton pump inhibitors in peptic ulcers and gastroesophageal reflux disease, after a single study that showed a marginal risk of gastrinoma.^12^

The fifth point of criticism against the results from the WHI is that it is just a single clinical trial. It would here be appropriate to recall the results from Heart and Estrogen/Progesten Replacement Study (HERS)^13^ and Estrogen Replacement Atherosclerosis (ERA).^14^ These two earlier clinical trials investigating secondary prevention did not show any advantage for HRT, in the same way as shown by WHI.

The sixth point relates to the fact that the WHI revealed that HRT has a protective role in relation to avoiding hip and vertebral fractures. Such a role cannot, however, constitute a reason for prescribing HRT, because osteoporosis can be managed in a more efficacious manner using other medicines such as bisphosphonates and raloxifene.^15,16^

The seventh question relates to the ages. The feasibility of a clinical trial using younger women is very low because of the high cost of the long-term follow-up that would be needed in order to wait for cardiovascular events and breast cancer to appear among this low-risk population. For this reason, the investigators in the Women's Health Initiative study chose the age group that they did.

The eighth question relates to subclinical atherosclerosis. There is no consensus regarding the best way of evaluating the association between some atherosclerosis markers (carotid Doppler for the thickness of the medial-inner layer or electron beam for coronary calcification, among other methods) and clinical endpoints (e.g. myocardial infarction, coronary heart disease mortality). Recently, a multicenter and multiethnic evaluation was launched to verify the real meaning of subclinical atherosclerosis, by means of a prospective study.^17^ However, out of the 16,000 women participating in the WHI study, divided into random groups, it is reasonable to suppose that those with subclinical atherosclerosis would be equally distributed between the two branches of the study.

In the light of this new evidence, it has been fascinating to see how fast the North American Menopause Society gave up recommending HRT for primary prevention of coronary disease.^18,19^ I can add that the results from recent studies like HOPE^11^ and HPS^20^ have brought real benefits to women at high risk of cardiovascular disease. With regard to healthy women, there is nothing more appropriate than recommending diets that allow suitable body weight to be maintained and moderate exercise for preventing cardiovascular disease.^21^ If HRT has brought much more hype than hope to women's health, on the other hand changing patients’ lifestyles and prescribing anti-converting enzyme inhibitors^11^ and statins^20^ for those at high cardiovascular risk are measures that must be strongly recommended.

Finally, there is a question of the highest importance, which doctors need to face up to directly: the enormous pressure from the pharmaceutical industry^22^ for creating diseases and "fountain of eternal youth" propositions, such as the one that has been built into the HRT proposal. Haven't we all to some extent been innocently co-opted by such interests?
